# Nursing Section

**Published:** 1904-03-26

**Authors:** 


### The Hospital.

**Nursing Section.**

Contributions for this Section of "The Hospital" should be addressed to the Editor, "The Hospital" Nursing Section, 28 & 29 Southampton Street, Strand, London, W.C.

No. 913.—Vol. XXXV. *SATURDAY, MARCH* 26, 1904.

### Notes on News from the Nursing World.

### QUEEN ALEXANDRA'S IMPERIAL MILITARY NURSING SERVICE.

Several new appointments and a number of important changes have been made in Queen Alexandra's Imperial Military Nursing Service:—Miss G. M. Richards has been appointed matron and temporarily posted to Woolwich; Miss W. M. Jay, Miss F. M. MacGregor, and Miss M. MacGregor have been appointed staff nurses to serve at Cambridge Hospital. Changes of duties are announced in reference to the following matrons: Miss A. L. Cox, Connaught Hospital, Aldershot, to Shorncliffe; Miss M. C. Knox, R.R.C., Aldershot to Dover; Miss S. E. Oram, R.R.C., Dover to the Cambridge Hospital, Aldershot; Miss G. M. Payne, R.R.C., Dover to Egypt; Miss H. W. Reid, Aldershot to South Africa; Miss E. A. Wilkinson, Woolwich to South Africa. The changes of stations among sisters are: Miss A. Fitzgerald, Miss E. C. Humphreys, Miss M. Pedlar, and Miss L. A. Rideout, Woolwich to Cambridge Hospital, Aldershot; Miss C. Anderson, Canterbury to South Africa; Miss M. E. Harper, R.R.C., Shorncliffe to South Africa; Miss R. Osborne, to Aldershot; Miss H. Suart, Netley to Cambridge Hospital, Aldershot; Miss M. M. Tunley, Caterham to Cambridge Hospital, Aldershot; Miss L. W. Tulloh, R.R.C., Alton to Canterbury; and Miss M. Wright, Cork to South Africa. Among staff nurses, Miss S. Smyth and Miss A. L. Walker go from South Africa to Cambridge Hospital, Aldershot; Miss A. R. F. Auchmuty and Miss S. K. Bills from Netley to Cambridge Hospital, Aldershot; Miss C. Mackay, from Gosport to Woolwich; Miss C. G. Stronach, from Fermoy to Woolwich; and Miss A. A. Wilson, from the Royal Herbert Hospital, Woolwich, to the Royal Arsenal, for temporary duty.

### THE QUALIFICATIONS FOR AMERICAN ARMY NURSES.

The Surgeon-General of the United States has opened in his office an "Eligible List of Volunteer Nurses." The names of acceptable graduate nurses who are willing to serve in time of war or national emergency will constitute this list, but we mention it chiefly because it sets forth the qualifications. Applicants, it is stated, must have graduated from a training school for nurses which gives a thorough professional education, both theoretical and practical, and which requires at least a two years' residence in an acceptable general hospital, of not less than fifty beds. Graduates from special hospitals, from asylums for the insane, or private sanatoria, will not be eligible unless their training has been supplemented by not less than six months in a large general hospital. It will be seen that the qualifications fall below those required in the case of members of Queen Alexandra's Imperial Military Nursing Service.

### THE NEW MATRON OF THE ROYAL PRINCE ALFRED HOSPITAL.

We are officially informed by the Agent-General for New South Wales that the selected candidate for the appointment of matron of the Royal Prince Alfred Hospital, Sydney, is Miss Mabel Newill, now home sister at the Hospital for Consumption and Diseases of the Chest, Brompton. Miss Newill's nursing career entirely justifies the choice which has been made. Trained at King's College Hospital from February 1894 to February 1897, she proceeded, soon after receiving her certificate, to obtain training in midwifery at Manchester Maternity Hospital, and in January 1898 was awarded the certificate of the London Obstetrical Society. She then became night sister at the Seamen's Hospital, Greenwich; and subsequently ward sister at University College Hospital, remaining there until July 1902. From Novemb[illegible] 1902 until the present time she has occupied the position which she now vacates. It cannot be doubted that her varied [illegible]ience at some of the leading schools, and the special knowledge which she must have acquired, will prove of great value in the extremely responsible office to which she has been elected from among a large number of candidates. We congratulate Miss Newill upon the fact that in ten years she has passed through all the grades of the profession and reached the front rank.

### A NURSES' HOME FOR UNIVERSITY COLLEGE HOSPITAL.

Thanks to the generosity of Sir Donald Currie and three of his daughters, University College Hospital will soon have a suitable Nurses' Home on the site which already belongs to the institution. Sir Donald has specifically given £20,000 in order to provide it, and £2,500 has been contributed by Mrs. Mirrielees, Mrs. Molteno, and Mrs. Wisely for the furnishing and provision of a library and piano. Sir Donald Currie has also signified that his daughters are willing to render any assistance in their power for the benefit of the nurses.

### GOLD MEDAL FOR A MATRON.

An interesting little function took place at the annual meeting of Governors of the London Temperance Hospital last week. This was the presentation of a gold medal to the Matron, Miss A. I. Richardson, in recognition of her services during the past 13 years. Miss Richardson already possessed the bronze medal given at the end of two years, and the silver one representing the completion of the three years' course of training; the gold medal is, however, only given for distinguished service, and the present is the fifth instance in which it has been received by a matron of the hospital, the last recipient being the late matron, Miss Lucas. The design is similar to that of the bronze and silver medals, viz., a St. Andrew's cross with a raised centre bearing the initials L.T.H., surmounted by the words, "For His Sake," while on the obverse is an inscription stating that the presentation is made in appreciation of services to the hospital from September 1901, including a period of seven years as Sister, and of two years and four months as Matron.

### AN ERRONEOUS ASSUMPTION.

Last week the matron of one of our best-known asylums complained that in a recent issue we had made some remarks which were likely to have the effect of discouraging asylum nurses, and, as another correspondent this week appears to share her views, we have endeavoured to see in what way we have exposed ourselves to this reproach. Our correspondents seem to base their criticisms upon the assumption that we suppose that the dismissed attendants at Tooting Bec Asylum are of a representative class, whereas we distinctly endeavoured to differentiate them. For this we had the best possible justification in a statement made to our representative, that the Tooting Bec Asylum was unlike any other asylum in the country, and that in consequence of the nature of the work it was difficult to obtain, as juniors, the services of very highly educated women. At Tooting Bec Asylum all the patients are imbeciles and usually old and helpless. The attendants have also to do the whole of the ward work. Even at an institution for aged imbeciles, however, we feel strongly that the nurses should enjoy the full advantages of training and education. We believe, too, that this is only a question of time, and, meanwhile, we most gladly acknowledge that at the leading mental asylums there are already a number of nurses who need not fear comparison on the score of ability or training with hospital nurses.

### A HOME FOR THE CITY OF LONDON CHEST HOSPITAL.

The need for better accommodation for the nurses of the City of London Chest Hospital, Victoria Park, has been long recognised, and within the last few months a start has been made by the Committee. It is estimated that the new building, in which eventually 40 sisters and nurses will be housed, will cost some £7,000, including furnishing and equipment, and it is proposed to hold a meeting at the Mansion House early in the summer with the object of raising the necessary funds. The Home is designed in sections, of which one, containing 30 bedrooms and the heating apparatus, lavatories and baths for the whole, has been commenced. The sisters' apartments, nurses' sitting-room, and about 10 bedrooms, will be left to the last, unless the amount wanted to complete the block is forthcoming. Details as to furnishing are not yet decided upon; there will, however, be an underground passage way to connect the block with the hospital, in which the present sitting-room will be converted into the nurses' dining-room, with a dinner lift from the kitchens below. As the nurses are at present housed in close proximity to the wards, while three of the cubicles share a window between them, and can only be used when absolutely necessary, in order to accommodate the staff, it must be acknowledged that the new home is badly required.

### A GLOOMY PROSPECT AT WORCESTER.

At the annual meeting of the Worcester City and County Institution for Trained Nurses, the chairman referred to the serious financial position which the report for last year disclosed. There was a decrease of over £100 in the amount earned in nursing fees, the subscriptions had fallen off, and the expenditure had not diminished. Altogether, the loss was £272 4s. 7d., and consequently, the report stated, the balance of £90 in hand at the beginning of the year had been absorbed as well as the £150 on deposit, while the banking account was overdrawn to the extent of £41. As some of the speakers said, the reason for the reduction of the amount earned in nursing fees might be a decrease of sickness; but a nursing institution ought to cut its coat according to its cloth, and if fees bring in less, the expenditure should also be less. There were upwards of 30,000 visits paid to the sick poor, but here, again, we are afraid that the decline in the subscriptions to the institution is due to the erroneous idea that the fees paid to the private nurses go far to cover the cost of the district work.

### UP-COUNTRY NURSING IN INDIA.

We so frequently receive inquiries about the work of the Up-Country Nursing Association that our readers may be glad to hear any details of actual experience. A sister, who is now engaged at Bareilly, says that the nurses are most happy and well cared for. In the home, breakfast is not served until 10 a.m., but tea and toast are provided at 6.30 or 7, and exercise in the open air follows. As there is constant sunshine and warmth during the day in winter, with roses in full bloom, she appreciates the climatic conditions, and altogether she seems to find her duties very congenial.

### NURSES AS PRIVATE DETECTIVES.

A Bill has been introduced in the Assembly at Albany, New York, for the purpose of amending the code of civil procedure so as to include trained nurses among privileged witnesses, such as physicians and clergymen, who are not compelled to disclose information obtained while attending a patient. It appears that the object of this measure is twofold—to protect nurses, and also to prevent them from being employed as detectives. This is because several nurses have not only been engaged in America as detectives, but have lately given evidence in their dual capacity in important legal cases. That there should be an agitation for legislation on the question shows that there must be many persons on the other side of the Atlantic who have had some very unpleasant experience in the employment of nurses.

### A TOUCHING INCIDENT AT SHOREDITCH.

The annual report of the Shoreditch and Bethnal Green District Nursing Association, presented at the meeting this week, is interesting and encouraging reading. During the past 12 months 1,630 cases were nursed and 38,965 paid—thanks to the Queen's Jubilee Institute, who are paying all the expenses of seven probationers. A grant from the City Corporation of £52 10s. enabled the Association to close its year with a balance in hand. In the earlier part of the year the committee made a new effort for raising funds by placing collecting boxes in various shops and other places of business. At the present time 50 boxes are out, but it is hoped to double the number. They are emptied quarterly, and so far £13 11s. 6d. has been added by this means to the funds. A touching incident was told the superintendent the other day. Every Saturday night a man enters a shop where a box is placed, without saying a word, puts twopence into it, and disappears quickly. He is known to be the son of a patient who was nursed from the Home a little time ago, and as he is evidently too poor to send a donation, he makes use of a box to show his gratitude. The incident is in itself an argument in favour of the extension of the box experiment.

### AN ADMIRABLE SUGGESTION AT SHREWSBURY.

At the annual meeting of the members of the Shrewsbury Victoria Nursing Association, which has now been established six years, a very satisfactory report was read and adopted. The total receipts amounted to £229 16s. 3d., and the expenditure to £194 5s. 9d., the balance in hand exceeding that of 1902. In spite of this result, however, there has been a slight falling off in subscriptions, and one of the lady members of the Committee accordingly suggested that all interested in the work of the Association should make an effort to obtain one new subscriber. This is an excellent practical proposal which is worth almost any amount of talk, and we do not doubt that if it is literally acted upon, many names will be added to the list of subscribers before the issue of another report.

### THE MATRONSHIPS OF DARENTH AND BELMONT ASYLUMS.

The administration of Darenth and Belmont Asylums has been under consideration for some time past by a sub-committee of the Asylum Committee of the Metropolitan Asylums Board, and at the meeting of the board on Saturday the sub-committee's report was submitted and adopted. With regard to the matrons of these institutions, the committee recommended that in the case of Darenth Asylum, the present headmistress, Miss Hoatson, should be placed on trial for a period of six months as headmistress and matron, and that for her services in that temporary or probationary appointment should be recompensed at the end of six months by a payment of £25. This, in addition to Miss Hoatson's present salary of £100, is equivalent to remuneration at the rate of £150 per annum. As to the matronship of the industrial colony, with pavilions, the committee recommended that the present matron of the schools, Miss Penney, who is a trained nurse, should be transferred to that position, the present matron of the adult asylum, Mrs. Williams, being transferred to be matron of Belmont Asylum. The recommendations were agreed to.

### STARTING A NURSE IN THE RIGHT WAY.

It was decided some time ago by the trustees of the late Miss Hector, of Peterhead, to apply a part of the residue of the estate in providing a nursing institute for the town. Steps have now been taken to carry out the scheme. A representative executive committee, consisting largely of ladies, with several medical men as advisory members, has been elected, and the first action of the committee has been to appoint a nurse who has had three years' training at Aberdeen Royal Infirmary. While the services of the nurse are mainly intended for the poorer classes, provision is made for others who are prepared to contribute to the funds to be entitled to the services of the nurse as far as practicable. All ordinary applications for their services are to be made to the secretary in writing, and in cases of accident or emergency by the doctor attending the case direct to the nurse.

### THE DUTY OF TORQUAY.

"Greater public support needed," was the burden of the speeches made at the annual meeting of the Torquay District Nursing Association. The number of patients attended by the nurses during the year were 278, and of visits paid 14,766. The special canvass made at the suggestion of Mrs. Ranson brought in £160, and the total of subscriptions and donations for the year reached £408 4s. 6d. They require about £450 a year to keep the matron and four nurses, and as the report says "it would be a lasting disgrace to Torquay if such a valuable local charity should have to cease its work through lack of adequate support." There should not even be a risk of its work having to be crippled.

### SHORT ITEMS.

A complete Naval Red Cross detachment, with nursing sisters, has recently reached Port Arthur.—The Bedford Guardians have agreed to adopt a system of alternate day and night nursing. Hitherto there has been a permanent night nurse, but in future each nurse will go on duty for a month at a time.—Fourteen nurses of the Poplar and Stepney Sick Asylum have just been examined in cookery under the Technical Education Board of the London County Council, with the result that 13 out of 14 received first-class certificates.

### The Nursing Outlook.

"From magnanimity, all fear above;
From nobler recompense, above applause,
Which owes to man's short outlook all its charm.'

### NURSING IN GREAT BRITAIN.

### III.—THE EXISTING ORGANISATIONS.

THERE can be no question that the interests of the public and of the nurses alike suffer at present from the absence of a central authority which is sufficiently representative to entitle it to the confidence of all who train nurses, of the nurses themselves, and of the public. At the present time, as we pointed out last week, practically all hospitals, however large or however small they may be, take probationers for training. The consequence is that, however perfect or imperfect the training may be, each person who has entered for it in the end may, and as a matter of fact does, offer her services as a trained nurse, and endeavours to earn the same rate of remuneration. This is a very serious and increasing evil; for whereas it may be essential to the proper accomplishment of the work which sick people require to be done for them in varying measures in the time of illness, that there should exist differences in the period and quality of the training, and even in the relative efficiency of the individual nurse, it is a great injustice for the well-educated, highly trained and exceptionally capable nurse that she should be unable to have a hall-mark placed upon her efficiency which will enable her to receive the highest emolument to which she is undoubtedly entitled. The present state of things has been growing worse and worse year by year, and the time is approaching, if it has not even arrived, when the great nurse-training schools must put themselves at the head of a movement in favour of reform, or suffer a loss of prestige and allow the control of the central authority over British nursing, which it is essential to create, to pass into other hands than their own.

The existing authorities are the great nursing training schools, the committee of each hospital where nurses receive some sort of training, the Royal British Nurses' Association, the Central Hospital Council for London, the Matrons' Council, and the Association for Promoting the State Registration of Trained Nurses. There are, in addition, other special branches of nursing, provided for by the independent examination of the Obstetrical Society of London for Midwives, the Medical Psychological Society for Mental Nursing, the Central Inspectors Examination Board for sanitary science and hygiene, and the Incorporated Society of Trained Masseuses for massage. The work of these agencies has done much to improve the education of nurses in the special branches which they represent. A central Midwives' Board is now raising the status of midwives throughout the country.

Taking the first-mentioned authorities in order, we propose to deal with the great training schools in a separate article. The committees of smaller hospitals, although they may properly claim representation on any central board for organising nursing in Great Britain, cannot take effective action on their own initiative. The Royal British Nurses' Association and the Association for Promoting the State Registration of Trained Nurses, have each introduced a Bill into Parliament this session. The former association shows no sign of increasing influence,, from causes which are too well known to need repetition here, and the latter is a young organisation which is in no sense representative of British nursing to day—it is, indeed, but a child of the Matrons' Council. Anyone familiar with hospital nursing matters in Great Britain, who obtains a list of the members of the Matrons' Council and studies the names upon it, will see at a glance that it can lay no proper claim to speak with authority on nursing matters in England to-day. The Central Hospitals Council for London has hitherto consisted of 35 representatives of the medical staffs and committees of the 12 London hospitals having medical schools. It has just passed a resolution in opposition to any State registration of nurses, and is opposing the present Bills in Parliament. It is no doubt representative of the governing bodies and medical staffs of the twelve hospitals in question, but they cannot be said to represent in any real sense the training schools, for the Council has no women members, and without these its influence, so far as nursing is concerned, must, to say the least, be imperfect and incomplete.

This exhausts the list of the nursing organisations referred to, and it will be admitted that none of them can claim to adequately represent the nursing profession in Great Britain at the present time. Some of them are continually fighting each other in public, and all of them exist, in the first instance, to promote the special aims of the more active spirits which control their policy. For this very reason it is essential to the wellbeing of everybody concerned that a central organisation of nursing throughout Great Britain should be established upon a wide enough basis to include all interests without further loss of time. We must defer an account of the lines upon which such an organisation must be created, if it is to be made really effective for its purpose, to a subsequent article.

### Lectures Upon the Nursing of Mental Diseases.

By ROBERT JONES, M.D.Lond., B.S., F.R.C.S.Eng., M.R.C.P.Lond., Resident Physician and Superintendent of the London County Asylum, Claybury.

### LECTURE VIII.

*The Heat of the Body.*—However cold the outside temperature may be, and whether man lives within the frigid zone or in the tropics, the human temperature does not vary. It is always in health 98·4.° There is an accurate balance kept between the gain of heat to the body and its loss from the body. When the gain to the body through the assimilation of food is balanced by the waste material excreted the equilibrium of the body is maintained. The sources of heat to the body are physical and chemical—the resolution of energy into heat affording an example of the former, and oxidation of the latter. Heat is produced by the process of oxidation which goes on in every living tissue, especially in the muscles, which, when contracting, cause a chemical decomposition—a breaking down of complex chemical tissues and the formation of simpler waste bodies, such as carbon dioxide and water. The energy resulting from muscular contraction appears as the heat of the body. It is probable that there is no "thermic" or heat centre, and no special heat nerves.

The liver is a great producer of heat, for the proteids brought into it are broken down into urea and glycogen; the latter is deposited in the liver, but in certain diseases, or through the action of the nervous system, it can be specially called upon.

The heart also in its contractions produces heat, as well as driving the blood over the body.

In cold weather heat production is increased. There is increased muscular activity, and the vessels in the skin can be controlled by the vaso-motor nerves to contract and to diminish heat loss. Also there is less secretion of perspiration with reduced evaporation, and thus less cooling in consequence. To prevent loss of heat, use is made of the fact that dry air is a bad conductor of heat, and layers of clothes are worn to ensure the presence of layers of air. Clothes which entangle the air in meshes, such as flannels, are thus warmer than closely woven linen. Damp air is an excellent conductor of heat, and it is in this way that damp air or wet or moisture is so injurious; for the heat of the body is insensibly carried away, and the temperature unduly cooled. Dry heat or dry cold is healthy and stimulating and invigorating; but it is the reverse with moisture, as anyone knows who has experienced the effect of damp days in summer or winter. Some of the heat of the body is lost in warming the clothing, and it is thus conducted away (conduction). The air, especially when wet, takes some heat and the rest is radiated from the body also into the air (radiation).

Of the total heat of the body at any one time, one-third is used to raise the temperature of the food, air, and drink taken into the body or lungs. Five-sixths of the effect of food is used in maintaining the temperature or the heat of the body, and one-sixth to produce energy. Fat food is calculated to be just twice as valuable for producing heat as proteids or starchy foods (carbohydrates). It is thus that fat is easier and better taken in cold weather. It is not known how the excess of proteid or meat food is stored up in the system—other than being incorporated in the living tissues.

As to supplying the energy and heat of the body, something definite should be known by the nurse in respect of the amount and varieties of food. The anatomical structure of man adapts him to select his food from both the animal and vegetable kingdoms. His teeth and the whole alimentary canal—the stomach and the intestines—are constructed for a mixed dietary; and the following divisions into five categories sums up the classification of foods, without one or the other of which, for any lengthened period, ill-health results.

1. *Water*, which enters more or less into the composition of every kind of food, is a daily requirement. The average quantity necessary for a healthy full-grown adult is estimated to be about three pints.

2. *Fats*, such as are contained in butter and the cream of milk, meat fat, suet, and lard. All these are of about equal nutritive value. It is stated that tuberculosis and typhoid fever may be transmitted through butter, and some recommend the process of clarification by boiling, in order to destroy any possible disease-germs. Vegetable fats, such as olive oil and the oil from poppy-seeds and nuts, are quite equal to animal fats in food value. I am of opinion—but I do not know how far this is shared by others—that the general dietary of the insane needs more fat than does the sane, and I am in the habit of supplementing this with cod-liver oil or dripping, and often to great advantage.

3. *Starches* and *Sugar*, as existing in potatoes, rice, fruit, beetroot, and cereals generally, are of good food value, especially when the starch of grain is used as porridge, and taken with milk. The nutritive value of fruit depends mainly upon the amount of sugar contained, but nuts, almonds, chestnuts, and other proteid and fat-containing seeds are also of great nutritive value.

4. *Albuminous* or *Nitrogenous* foods, or proteids, which all contain the elements of carbon, hydrogen, oxygen, and nitrogen, with some sulphur and phosphorus, constitute the greater part of such foods as lean meat, white of egg, the casein of cheese or milk (which also contains some fat). The gluten of flour and oatmeal are also examples of proteid foods. The nutritive value of cheese has been estimated to be two or three times as much as meat. Of vegetables, peas, beans, and lentils contain the greatest amount of proteid or albuminous material, and potatoes the least; they are, however, the cheapest form in which starch can be obtained, but they require fat and proteid to form a good sustaining meal. The legumes—peas and beans—are of great nutritive value when taken with fat, as in the country meals of "beans and bacon."

5. *Salts* of various kinds, such as common table-salt, lime, phosphates, potash, and magnesia, also enter into the composition of food, being required in the formation of the blood and tissues. Green vegetables are not of any great nutritive value, but they are wholesome in so far as they are convenient vehicles for conveying salts. The want of these is the cause of scurvy. In the last stages of general paralysis of the insane the food must always be finely minced; and not only must the meat be minced, but also the potatoes and green vegetables. The latter are a very necessary ingredient in the dietary of those patients ordered to be on "soft" food or "slops." Food varies much in the relative proportion of the different ingredients enumerated. Milk alone contains some of all of them. It is an ideal food for the insane, who often refuse ordinary food owing to delusions of poisoning. Cases of insanity in adolescents can take milk in large quantities. I have had a young patient taking ten pints of milk per day. Milk is richer in summer than in winter, in the evening than in the morning, and so is that last drawn than the first. Skimmed milk is a very good cheap food, but the poor have a prejudice against it. Buttermilk—that which is left after churning the whole milk and separating the butter—is also an excellent food, the casein having been well divided. Buttermilk is more digestible than skimmed milk. Milk is objected to on the ground that it forms a good nutritive medium for the growth of germs, and it may readily be contaminated and give rise to typhoid, scarlet, or typhus fever, and cholera. When not cleanly kept, from a bacteriological point of view, it causes diarrhœa, especially in children. For this reason such germs as cause the diseases mentioned, as also probably the bacillus tuberculosis, should be destroyed by sterilising—*i.e.* using milk only when boiled or raised to a temperature above the boiling-point in a specially adapted steriliser.

Meat when boiled gives up to form the broth most of its salts and extractives, but it still contains the proteids, and has suffered no appreciable loss as a nutritive agent. On the other hand, the broth and also the meat extract have little nutritive effect, but they have a great value as a stimulant. Roast meat, and meat which has been smoked, pickled, or tinned, contain all the nutritive elements, and when made aromatic form important accessories to the food, stimulating the mucous membrane to increased secretion; but there is danger from tinned foods as the food may be improperly selected, in consequence of which the protoplasm of the meat undergoes chemical change, resulting in the production of poisonous animal alkaloids, called *ptomaines*, which are similar in chemical composition to the poisonous vegetable alkaloids, such as aconitia, strychnia, morphia, and others. Almost all kinds of tinned meats are especially preserved by the addition of chemicals, such as formal, boric and salicylic acid; and it is well known that salicylic acid is closely allied in its chemistry to carbolic acid, which is an active and powerful poison when undiluted.

A great factor to the individual in the nutritive value of the different meats is their digestibility. Some persons are unable to retain or assimilate pork, veal, rabbit, or other meats, and a further important consideration is the mode of preparation. Danger to health may arise from meat when it is undercooked, for the trichinæ in pork and the larvæ of tapeworms in beef may always develop in the human intestine, with serious results. Animals may also be tubercular—that is, their flesh may contain the bacillus of consumption; but the supervision and official inspection by medical officers of health, under public control, ensures some protection, and disease through the medium of meat, when properly cooked, need cause no alarm. The special parts of animals, such as the liver, pancreas (popularly known as the "sweetbread"), and the kidneys are not of such nutritive value as the fleshy or muscular part, but they are often enjoyed by the sick as delicacies. Fish is of nearly equal value to the flesh of animals as a food, and is much appreciated as a change of dietary by sick persons. Care should be exercised in respect of shellfish, as serious cases of typhoid fever have been traced to oysters, mussels, cockles, and other varieties through contamination of the water of rivers or estuaries in which they grow, or even the water in which they are kept in shops pending their disposal.

Eggs, when soft boiled, constitute an easily digestible food, and the white, which forms two-thirds of the egg, is chiefly water, but it contains albumen and another proteid called globulin which are very nourishing. The remaining one-third—the yolk—contains double the amount of albumen and also a fourth of its volume is fat. When beaten up with milk an egg forms an excellent food; but, for invalids reduced through alvine discharges and with a low vitality and feeble digestion, albumen water (made by mixing the white of egg with water, and sweetened) is greatly appreciated as a food and beverage. The white of egg, when boiled, becomes hard, and forms an impenetrable barrier to the action of the gastric juice—hence its indigestibility.

Certain articles used with food, such as tea and coffee, can only be regarded as stimulants; and these, together with alcohol, often cause widespread harm when taken too frequently and in too great quantities. Much could be said upon the question of stimulants and luxuries; but with regard to alcohol, it is so subtle and overpowering in its influence and it is so easy to use it immoderately, and it is moreover of so low and indirect a nutritive value, that it has no claim to be considered as a food. It is further much adulterated by the addition of noxious substances and its effects are so insidious, evil, and destructive to all the organs of the body, that catarrh of the stomach, fibrous growth in the liver, and degeneration of the brain follow its use. It should therefore never be encouraged as a beverage. It should be used solely as a drug, and only under medical advice.

### State Registration of Trained Nurses.

### A REPLY TO THE ANTI-REGISTRATION MANIFESTO.

The signatories of the manifesto against the State Registration of Nurses, which appeared in the columns of THE HOSPITAL of the 19th inst., have failed to grasp the aim and purpose of the Bill introduced into the House by the Association for promoting the State Registration of Nurses.

The central purpose of this Bill is the organisation of the profession by the establishment of a representative Nursing Council, to whom all questions of training, discipline, and other professional matters will be referred, and by whom a register of trained nurses will be kept.

The first duty of the Council (on which the public, the medical profession, matrons, and nurses must be represented) will be to determine what is the minimum training and experience necessary to constitute a fully-trained nurse. Once such a standard established, nurses who have received this minimum training in hospitals or groups of hospitals approved by the Nursing Council, will be entitled to register, and will receive a certificate which will be recoverable by the Council in cases of moral or professional misconduct. It is not suggested that registration should depend upon an "examination," or that the methods of training should differ materially from those now in force in our leading hospitals. Matrons of hospitals in which nurses undergo training will have precisely the same opportunities of judging of the moral qualities of their nurses as they have under the present system; and it is not apparent why that side of a nurse's equipment should receive less consideration under a system of registration than it does at present. State registration will in no way prevent employers from making further inquiries into the personal qualifications of a nurse, but in the innumerable cases where, owing to death or changes in matronships, no such references are possible, the employer will have an assurance that the registered nurse has gone satisfactorily through a recognised training-school, and has been considered eligible for registration by her matron. It would be needless to anticipate that hospitals will keep in their employ and recommend as candidates for registration women of unsuitable character and disposition. Nurses will therefore not be registered on their technical qualifications alone, inasmuch as the training-schools will certainly in every instance be required to testify that candidates possess the necessary personal qualities.

The public are in a position to judge for themselves whether or not a woman is a desirable member of their households; they have not the expert knowledge to enable them to estimate her professional qualifications.

It must also be borne in mind that many nurses do not work in connection with either hospital or agency, and that the public has no guarantee of the efficiency and impartiality of particular agencies. Some maintain an adequate standard of professional efficiency; others are run purely as commercial speculations in the interests of the proprietors.

Under a system of registration the public would obtain their nurses from institutions which guaranteed only to supply registered nurses, and they would then not pay full fees for hospital probationers still in training, or for half-trained, inefficient women farmed out as a commercial speculation for the pecuniary advantage of a middleman.

The advocates of registration deny that the present want of organisation, and the absence of any definite test of professional efficiency, is a guarantee of the possession by nurses of superior moral qualities, and they also contend that—however valuable personal qualities may be—it is through technical skill alone that a nurse is rendered valuable in cases of serious illness. The possession of the highest character and the best intentions would not enable an ignorant woman to arrest the hæmorrhage of a wounded artery or adjust a bandage carefully and skilfully. On the other hand, education and technical training tend to cultivate "observation, sympathy, cheerfulness, and self-control," and the highly-skilled usually acquire that professional zeal or devotion to a patient's interests which is likely to be better directed and more effective than any amount of native, but unskilled, kindness and sympathy.

It is not suggested that there should be a "uniform training," but that a certain minimum standard of training should be laid down, anything less than which cannot be regarded as complete or sufficient.

Non-registered nurses will be free to work for hire, but will be forbidden to claim the title of "registered nurse," and it is maintained that registration, by giving a definite status to the fully-trained nurse, and by enabling the public to discriminate between the quality of the services offered, will attract a good class of women into the profession, and will lead to a better sub-division of labour, and to greater economy of nursing effort.

The State register would in no way "lower the status of the best nurses," for, although every nurse on the register would have reached a certain minimum standard of efficiency, those who undertook further courses of study, or obtained experience in different departments of nursing, would have these facts duly notified in the register, and this would result in the creation of a higher order of nurses, to whom higher fees might be paid, and by whom the prizes of the profession would naturally tend to be filled.

The opposition to registration comes from Hospital Committees—the employers of nurses—and from some matrons in the service of these committees, who have ranged themselves on the side of their employers. It is unnecessary to point out that the interest of employer and employed are not always identical, and both need safeguarding. There is also considerable prejudice in the minds of Hospital Committees against any proposals which would establish a central board, and thus diminish the independence of their nurse-training schools.

At present the training schools have absolute power to regulate the hours of work and the conditions of labour of their employees. In some hospitals the position of the probationer is inferior to that of the domestic servant, who at any rate is entitled to a month's notice. Neither have women who enter the training-schools any guarantee at present that they will receive a thorough training in return for the three years' service to which in most cases they bind themselves on the understanding that they will receive an efficient training.

These training-schools are at the present time a law to themselves. There is no minimum standard to which they must conform before they are entitled to rank as training-schools, and, while some give due consideration to the interests of their pupils, others—as is only natural—consider their own convenience first.

As an efficient nursing service is a valuable "national asset," it is imperative that nurse-training schools should give proof of their qualifications before their recognition as educational authorities. This is imperative in the interests of the nurses and of the public.

*Signatories to the Appended Reply to the Anti-Registration Manifesto.*

Louisa Stevenson: President of the Society for the State Registration of Trained Nurses, Member of the Board of Management of the Edinburgh Royal Infirmary. Ethel Gordon Fenwick: President International Council of Nurses, Member of Distinguished Order of the Greek Red Cross. Isla Stewart: Matron and Superintendent of Nursing St. Bartholomew's Hospital, President Matrons' Council of Great Britain and Ireland, President League of St. Bartholomew's Hospital Nurses. Margaret Huxley: President Irish Nurses' Association. Mildred Heather-Bigg: Matron Charing Cross Hospital. Eleanor C. Barton: Matron Chelsea Infirmary, President Chelsea Infirmary Nurses' League. G. A. Rogers: Matron the Leicester Infirmary, President League of Leicester Infirmary Nurses. M. Mollett: Matron Royal South Hants and Southampton Hospital, President Royal South Hants Nurses' League. Margaret Breay: Vice-President League of St. John's House Nurses, Hon. Secretary Matrons' Council. Helen Munro Ferguson: Vice-President Society for the State Registration of Trained Nurses. Fanny Lumsden (of Belhelvie): Vice-President Society for the State Registration of Trained Nurses. Flora C. Stevenson, LL.D.: Chairman of the Edinburgh School Board, Vice-President Society for the State Registration of Trained Nurses. Ruth Howan: Member of the London School Board, Vice-President Society for the State Registration of Trained Nurses. May Tennant: Formerly H.M. Superintending Inspector of Factories, Vice-President Society for the State Registration of Trained Nurses. Clara Belasyse Myers: President Millom Nursing Association, Vice-President Society for the State Registration of Trained Nurses. Madelaine Greenwood: Late Hon. Secretary Society of Women Journalists, Vice-President Society for the State Registration of Trained Nurses. A. M. MacDonnell, R.R.C.: Lady Superintendent Richmond Hospital (Dublin), Member Irish Nurses' Association, Vice-President Society for the State Registration of Trained Nurses. J. Kildare Treacy: Lady Superintendent City of Dublin Nursing Institution, Member Irish Nurses' Association, Vice-President Society for the State Registration of Trained Nurses. S. E. Hampson: Member Irish Nurses' Association, Vice-President Society for the State Registration of Trained Nurses. Euphemia Ross: Matron Western Hospital (Fulham), Delegate Matrons' Council, on Executive Committee

Society for State Registration of Trained Nurses. Rosamund Grace Hayward: Delegate League of St. Bartholomew's Hospital Nurses, on Executive Committee Society for State Registration of Trained Nurses. Laura P. M. Baker: Delegate League of St. John's House Nurses, on Executive Committee Society for State Registration of Trained Nurses. Eleanor Richardson: Matron Stockport Infirmary, Local Hon. Secretary for Cheshire. Frederica M. L. Groves: Hon. Secretary for Gloucestershire. Henriette C. Poole: Matron East Lancashire Infirmary, Blackburn. K. Macintyre: Matron Royal Albert Edward Infirmary, Wigan. Margaret F. Mulvany: Matron Bolton Infirmary, Hon. Secretaries for Lancashire. B. L. Greenlaw: Matron Allt-yr-yn Hospital, Newport; Hon. Secretary Monmouthshire. Helen L. Peatse: Superintendent of Nurses North Staffordshire Infirmary, Hon. Secretary Staffordshire. Annie Barling: Matron Kidderminster Infirmary, Hon. Secretary Worcestershire. F. Cicely Gower: Hon. Secretary Brighton. Rachel Foley: Matron Royal Hospital (Richmond), Hon. Secretary Surrey.

### Choosing the New Matron of the Royal Prince Alfred Hospital, Sydney.

In connection with the appointment of Miss Newill as matron of the Royal Prince Alfred Hospital, Sydney, we understand that before Professor Anderson Stuart, M.D., chairman of the hospital, left Australia for Europe, he, in conjunction with the secretary, Mr. Epps, and the retiring matron, Miss McGahey, drew up the following memorandum.

Royal Prince Alfred Hospital.

*Memos* re *Matronship.*

*Age.*—Should be young and energetic, preferably from 30 to 35.

*Physique.*—Should be strong and of good presence. Applications from candidates of markedly nervous temperament should not be entertained.

*Status.*—Should be unmarried.

*Experience.*—Should be a person of recent experience in a large up-to-date hospital, and should bring with her modern ideas and methods, and not have to be dependent for her knowledge upon what she could gain in the hospital after arrival. If possible previous experience should have been gained in two large general hospitals. She should have a considerable knowledge and experience of housekeeping and not have been confined purely to nursing work. She should have had experience in the instruction of and delivery of lectures to nurses; should for preference be a nurse who has shown an interest as such outside her own hospital, as, for instance, by taking an active part in nursing associations, etc.

*Duties.*—While it is difficult to specify the various duties of the matron, which to a trained person will not need enumerating, it is considered desirable that the matron should act as far as possible as an overseer (in accordance with the rules) and leave the actual carrying out of details to her immediate officers. This should specially apply to the housekeeping, both of the hospital and the nurses' home. The matron will arrange her hours of duty according to the necessities of the work, but it is desired that she should have a due regard to her own health.

*Morale.*—It is desirable that the matron should have a high ideal as to her profession, and constantly aim at training her nurses up to the highest standard of practical efficiency and moral excellence, and with the idea before them that they will afterwards represent the hospital, whose reputation will be in their hands.

*Characteristics.*—The matron should be a woman of firmness of character and a good disciplinarian capable of governing nurses. But she should also possess tact and common sense, which indeed are more essential than much theoretical and nursing knowledge. It should be her aim to make the nurses regard her as a friend as well as look up to her as their governor.

### The Irish Nurses' Association.

The rooms of the Irish Nurses' Association, 86, Lower Leeson Street, Dublin, were formally opened on the evening of St. Patrick's Day, by the President, Miss Huxley, who spoke of the need for a central place where professional matters could be discussed, and where it was possible for provincial nurses to apply for information upon points affecting their work.

Mrs. Kildore Tracy, Matron of the City of Dublin Nursing Institution, next addressed the members, and urged them to take an intelligent interest in their own affairs, alluding to the Bill for State Registration of Trained Nurses now before Parliament. Mrs. Tracy emphasised the necessity for probationer nurses taking a special interest in this matter, as they were only beginning their careers, and were likely to be much more affected by legislation than those who had already been working for many years.

Miss Haughton, Matron of Sir Patrick Dun's Hospital, who had charge of the musical arrangements, then asked all present not to forget the social side of the matter, reminding the nurses of the many pleasant reunions they enjoyed in the days of the former Nurses' Club, which, she hoped, would be repeated now that they had such a pleasant meeting place.

Miss Helen Shuter, Matron of the Royal City of Dublin Hospital, also said a few words expressing her pleasure at the large and representative gathering of matrons and nurses present, and her hope that it might be productive of much benefit.

Tea was then partaken of, after which several members entertained the company with some delightful vocal and instrumental music. No fewer than 250 matrons and nurses found it possible to be present at this busy season.

### Everybody's Opinion.

### PORTRAITS AS PRESENTATIONS.

"A Staff Nur[illegible]e" writes: With regard to the proposal that min[illegible]ture portraits should be presented to officials who h[illegible]e done excellent work in hospitals, may I say that the fe[illegible]ling in our hospital is that not only should we like to see a faithful representation of our matron or home sister in the board-room or on our sitting-room wall, but I am sure that nearly all of us would be glad to give towards a portrait of our Chairman, whom we all know has done so much for us. Here so many would wish to subscribe that the amount contributed by each would be very small. It would be exceedingly interesting in the years to come if we could start, as it were, a little gallery containing those of whom we think the highest, and which in the years to come might grow into a most interesting collection.

### SIR WILLIAM BENNETT ON PRIVATE NURSING.

"A Dublin Nurse" writes:—I beg leave to say a few words more on this subject, particularly with reference to the further letter addressed to you by "A Matron" appearing in your issue of the 12th inst. With every personal courtesy, I desire to say that my sister nurses here—and I may add also our own matron—together with myself, did read her letter of 20th ult "very carefully." If the meaning of the letter were so "obvious" as "A Matron" thinks, her example of "two gentlemen" by way of explanation is surely quite superfluous as well as irrelevant. The illustration is also illogical—as, if one of the gentlemen referred to were "a fully qualified medical practitioner," and the other not, they could not be "equal with regard to educational advantages." The present discussion ("A Matron" does not stick to the text) is simply one raised by my letter, as an Irish nurse, inserted in your columns of 30th January last, after Sir William Bennett's statement that, in the relation of nurse to patient, "there is no such thing as 'decency or indecency.'" "A Matron" seeks to explain that what she wrote with reference to institutions with untrained matrons was not in any way disparaging to untrained matrons as women; but that, *in those institutions*, a nurse might sometimes, under exceptional circumstances, be in some doubt what is really right for her to do. Now, how possibly could the matter of her matron's training, or the want of it, cause "doubt" in the mind of a trained nurse—a "womanly woman" when away on a private nursing case? And, even assuming such a possibility, how would the "doubt" be set at rest by a matron, certainly not at the nurse's elbow if any question of decency, or otherwise, arose? After all, this a nurses' affair far more than a matron's, and it was to the former that Sir William Bennett addressed his remarks. I venture to think that the views I have already expressed in the correspondence on this subject carry the approval of very many in our profession, and it is pleasing to observe from your Editorial note that, owing to the interest it has excited, Sir William's address has been reproduced in pamphlet form, for the advantage of our sisterhood all over the world.

[The pamphlet to which "A Dublin Nurse" refers is published by the Scientific Press, Ltd., 28 and 29, Southampton Street, W.C., price 6d., post free, 7d.—ED. HOSPITAL.]

### ASYLUM NURSES.

"AN ASYLUM NURSE" writes: A recent unfortunate incident at Tooting Bec Asylum has been the occasion of the appearance in your columns of some remarks on Asylum Nurses in general which seem to be unjust to the class of nurse and almost uncalled for. No possible good can come of comparisons between two classes of nurses when these are made obviously with the intention of belittling the one class and lauding the other. No doubt it was from the "Hospital Nurse" that the cloud of ignorance and inability which enshrouded the nursing profession in the time of Dickens first began to pass, but it is unfair to assume that even yet it has not lifted from the "Asylum Nurse." In every class of beings there are to be found the good mixed with the bad, and I suppose asylum nurses cannot hope to be any exception to this rule, but I venture to think that nowadays the good are in a majority and that the minority is ever diminishing. Very many (and the number continues to increase) possess the nursing certificate of the Medico-Psychological Association of Great Britain and Ireland, and in all asylums the insane who are suffering from physical ailments, receive nursing which is both kindly and able and can undoubtedly court comparison with that of a general hospital without fear. It is true that we do not find in asylums the so-called "lady nurse" who enters a general hospital and undergoes training either as a hobby or for the pure love of doing good—asylum work under present conditions is too hard and too trying for such as these—but there are, nevertheless, to be found in asylums those whose work becomes a hobby to them and who minister lovingly to the victims of the most awful of the ills that flesh is heir to, who endeavour to cast some ray of hope into those tormented souls with whom they are in daily constant close contact from morn to eve. It requires some practical experience in dealing with the insane to know how trying this class of work is, and to understand how it is even much more necessary for this class of nurse than for the hospital nurse to possess all those higher attributes of humanity, tact, perseverance, forbearance, patience, gentleness, sympathy, and kindly manner, honesty, truthfulness, loyalty, etc., without which she cannot hope to command the respect and esteem of her patients—for it is possible to gain these even from the insane.

### Appointments.

CREWE ISOLATION HOSPITAL.—Miss E. Morgan has been appointed matron. She was trained at the Royal Infirmary, Newcastle-on-Tyne, where she was afterwards charge nurse, theatre sister, and night superintendent. She has since been matron-nurse of the Knight Memorial Hospital, Blyth, Northumberland, and matron of the Wallsend Joint Fever Hospital. She holds the Glasgow certificate for midwifery.

DARLINGTON FEVER HOSPITAL.—Miss C. Ethel J. Chaffer has been appointed matron. She was trained at the Clinical Hospital, Manchester, and the Queen's Hospital, Birmingham. She has since been charge nurse at the South Eastern and the Park Fever Hospitals, London; sister at the Fever Hospital, Portsmouth; sister at the Children's Hospital, Nottingham; matron (*locum tenens*) at the Throat Hospital, Golden Square, London; matron of the Western General Dispensary, Marylebone Road, London; and matron-in-charge of the Isolation Home in connection with the Infectious Diseases Hospital, Colchester.

KENSINGTON DISPENSARY AND CHILDREN'S HOSPITAL.—Miss N. Carruthers has been appointed matron. She was trained at the Cumberland Infirmary, Carlisle, and has since been sister in charge of the children's ward at Macclesfield Infirmary, and also sister at the Manchester Children's Hospital, Pendlebury.

MAIDENHEAD UNION INFIRMARY.—Miss Robbins has been appointed assistant nurse. She was trained at the Royal Boscombe Hospital, Bournemouth, by the Meath Workhouse Nursing Association.

MANCHESTER CONSUMPTION HOSPITAL, BOWDON.—Miss E. Folkes has been appointed matron. She was trained at the Manchester Children's Hospital, Pendlebury, and has since held the posts of ward sister, and home and theatre sister in the same hospital.

RODGETT INFIRMARY, ROYAL ALBERT ASYLUM, LANCASTER.—Miss Helen Rasey has been appointed nurse-matron. She was trained at the Royal Surrey County Hospital, Guildford, where she was subsequently charge-nurse and sister. She has since been night superintendent at the Southwark Infirmary, East Dulwich.

WINCHESTER COLLEGE SANATORIUM.—Miss Evelyn Montgomery has been appointed matron. She was trained at the Royal Hants County Hospital, and has lately been out-patient sister at the Victoria Hospital for Children, Tite Street, Chelsea.

### Novelties for Nurses.

BY OUR SHOPPING CORRESPONDENT.

### SPECIAL TERMS TO NURSES FOR COVENTRY CYCLES.

IN view of the opening of the cycling season at Easter, nurses will be glad to know that the well-known firm of Edward O'Brien, Limited, of Coventry, is offering cycles to them on special terms. Nurses who contemplate purchasing cycles may like to write for special quotations to Edward O'Brien, Limited, before coming to a decision.

